# EGFR and PDGFRA co-expression and heterodimerization in glioblastoma tumor sphere lines

**DOI:** 10.1038/s41598-017-08940-9

**Published:** 2017-08-22

**Authors:** Debyani Chakravarty, Alicia M. Pedraza, Jesse Cotari, Angela H. Liu, Diana Punko, Aushim Kokroo, Jason T. Huse, Gregoire Altan-Bonnet, Cameron W. Brennan

**Affiliations:** 1 Kravis Center for Molecular Oncology, Memorial Sloan Kettering Cancer Center, New York, NY 10065 USA; 2 Human Oncology and Pathogenesis Program, Memorial Sloan Kettering Cancer Center, New York, NY 10065 USA; 3 Brain Tumor Center, Memorial Sloan Kettering Cancer Center, New York, NY 10065 USA; 4 ImmunoDynamics Group, Programs in Computational Biology and Immunology, Memorial Sloan Kettering Cancer Center, New York, NY 10065 USA; 5 Center for Cancer Systems Biology, Memorial Sloan Kettering Cancer Center, New York, NY 10065 USA; 6 Department of Immunology, Weill Cornell Graduate School of Medical Sciences, New York, NY 10065 USA; 7 School of Medicine, University of California San Diego, 9500 Gilman Drive, MC 0602, La Jolla, CA 92093 USA; 8 New York Medical College, School of Medicine, 40 Sunshine Cottage Rd, Valhalla, NY 10595 USA; 9 NYU School of Medicine, 550 1st Avenue, New York, NY 10016 USA; 10 Department of Pathology, Memorial Sloan Kettering Cancer Center, New York, NY 10065 USA; 11 Department of Neurosurgery, Memorial Sloan Kettering Cancer Center, New York, NY 10065 USA

## Abstract

Concurrent amplifications of EGFR and PDGFRA have been reported in up to 5% of glioblastoma (GBM) and it remains unclear why such independent amplification events, and associated receptor overexpression, would be adaptive during glioma evolution. Here, we document that EGFR and PDGFRA protein co-expression occurs in 37% of GBM. There is wide cell-to-cell variation in the expressions of these receptor tyrosine kinases (RTKs) in stable tumor sphere lines, frequently defining tumor cell subpopulations with distinct sensitivities to growth factors and RTK inhibitors. We also find evidence for functional transactivation of PDGFRA by EGFR and EGF-induced receptor heterodimerization, both of which are abolished by EGFR inhibitors. These results indicate that GBM growth responses to targeted therapies previously tested in clinical trials are strongly influenced by the balance of EGFR and PDGFRA activation in individual cells, which is heterogeneous at baseline.

## Introduction

Primary glioblastoma (GBM) was the first tumor type selected by The Cancer Genome Atlas (TCGA) for broad genomic analysis powered by a large and homogeneous tumor sample set^1, 2^. While GBM is commonly described as molecularly heterogeneous, the most salient genotypic features emerging from TCGA and other large profiling efforts confirm a high degree of stereotypy and redundancy of Phosphoinositide-3-Kinase (PI3K) and Mitogen Activated Protein Kinase (MAPK) pathway alterations which occur in more than 80% of cases^1, 2^. Two-thirds of primary GBM harbor amplifications and/or mutations of receptor tyrosine kinases (RTKs), most commonly the Epidermal Growth Factor Receptor (EGFR, 60%) and Platelet Derived Growth Factor Receptor α (PDGFRA, 10–15%)^2^. While multiple studies have established a range of *in vitro* and *in vivo* sensitivities to inhibition of these mutations and their downstream pathways in GBM, clinical trials to date have failed to show consistent efficacy of any small molecule inhibitor as monotherapy^3–7^.

Nearly all activating RTK alterations in GBM involve amplification of the wildtype and/or mutant gene, typically in the form of extrachromosomal double-minute (DM) fragments that are heterogeneously distributed in tumor cells^2, 8, 9^. One or more mutant alleles may be expressed in addition to the wildtype, with a wide range of allelic ratios varying tumor-to-tumor and cell-to-cell^1, 2, 10, 11^. Downstream of the RTKs, alterations of PI3K inhibitory phosphatase, PTEN, and Ras negative regulator, NF1, have been found in 80% and ~20% of GBMs respectively, most commonly in a haploinsufficient state where cellular protein levels are sensitive to transcriptional and protein regulation^2, 12^. Together, these factors contribute to cell-to-cell variability in overall PI3K and MAPK pathway drive arising from variable gene dosage or protein expression of the most common GBM driver mutations. A phenomenon of increased growth rate and tumorigenicity in glioma tumorsphere subpopulations enriched for high EGFR expression has been previously noted^13^. However, the influence of cell-to-cell variation in RTK expression on cellular responses to stimulation and inhibition of RTKs and their downstream targets has not been systematically investigated and is an important issue for studies that rely on GBM tumor sphere lines for testing inhibitors of growth factor pathways.

Co-expression of multiple RTKs has been previously described in GBM and EGFR and PDGFRA are the most common pair co-activated (phosphorylated) in bulk GBM tumor even in the absence of amplification^5, 14^. There is a suggestion from genetic evidence that coactivation may be functional. At the DNA level, co-amplification of EGFR and PDGFRA loci has been observed in approximately 5–7% of GBM^8, 9, 15^. We previously demonstrated that co-amplification occurs in the form of a majority population of single RTK amplified cells (either EGFR or PDGFRA) along with minor populations harboring both EGFR and PDGFRA amplicons^9^. In such co-amplified tumors, nearly all mutations and DNA copy number changes outside of the amplified loci are shared among all tumor cells^8, 9^ suggesting that divergence of the single RTK-amplified cell lineages is a late event in tumor evolution. The simplest model explaining the observed pattern is that co-amplification of EGFR and PDGFRA is a driver event early in gliomagenesis prior to the tumor’s rapid expansion phase, and that subsequent tumor heterogeneity arises from random segregation of independent EGFR and PDGFRA double minutes in the daughter cells^9, 16, 17^. We therefore hypothesize that selection for EGFR and PDGFRA co-amplification within the same cell in early GBM formation may represent the adaptive synergy of unique signaling targets particular to each receptor and/or functional transactivation when both are expressed at high levels in the same cell. Indeed, we have previously reported PDGFRA phosphorylation by EGF in a coamplified tumor sphere line, which is blocked by EGFR inhibitors^9^.

In this study we characterize wide-ranging patterns of EGFR and PDGFRA co-expression among single cells sorted from patient-derived GBM tumor sphere (GTS) lines representing a selection of the most common genotypes, not restricted to co-amplified tumors. We demonstrate that EGF-stimulated EGFR-PDGFRA transactivation is prevalent and associated with receptor heterodimerization. Lastly, we find that single cell responses to both stimulation and inhibition of EGFR and PDGFRA are significantly modulated as a function of the expression levels of both receptors on each cell.

## Results

### EGFR and PDGFRA co-expression is prevalent in glioblastoma and occurs with significantly varied cell-to-cell distributions

**Figure 1:**
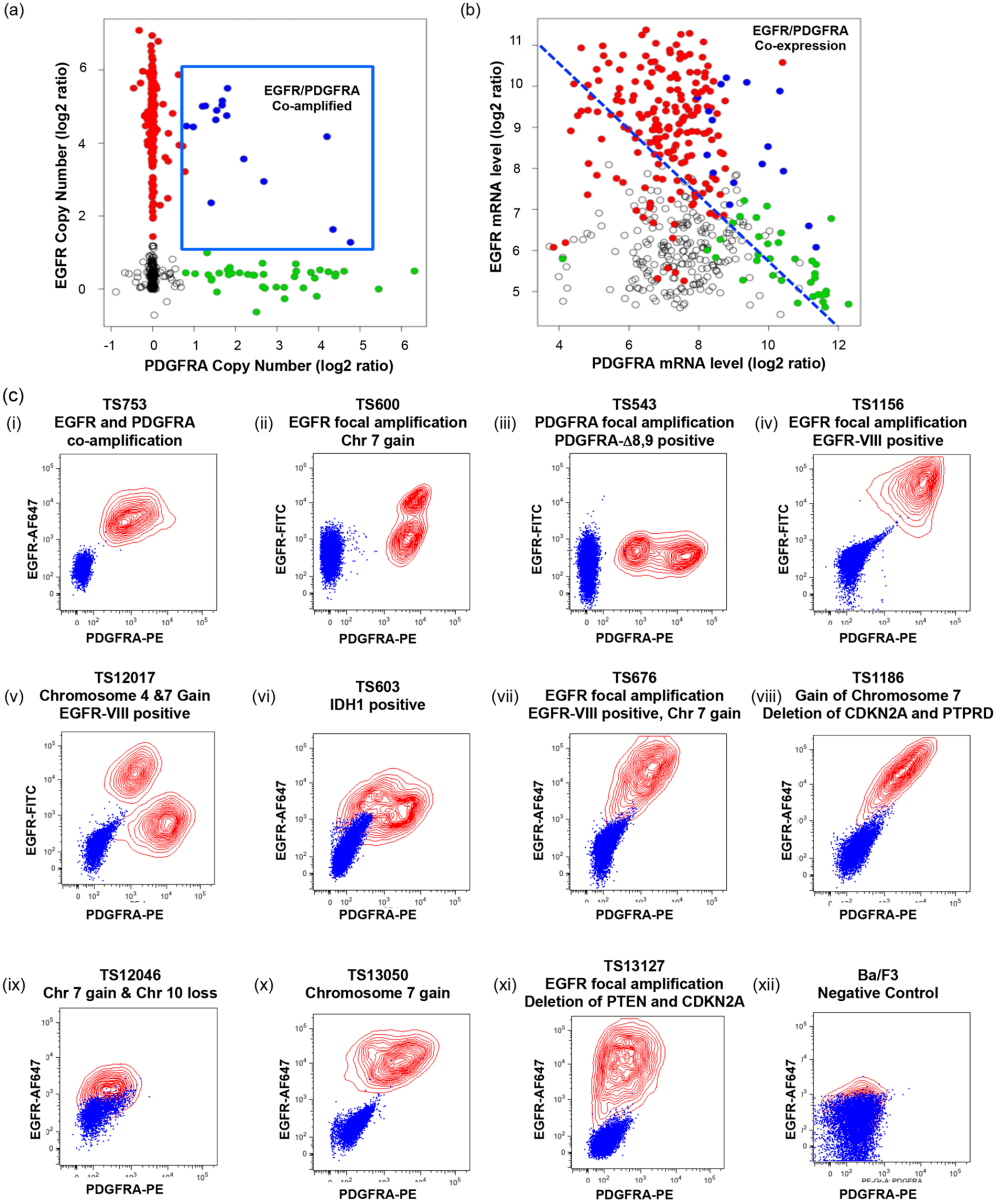
Co-expression of EGFR and PDGFRA in human glioblastoma is highly prevalent and exhibits diverse cell-to-cell distributions of EGFR and PDGFRA expression. (**a**) EGFR (y-axis) and PDGFRA (x-axis) copy number from 434 patient tumor samples of the TCGA glioblastoma cohort. 4% (16/434) of cases with clear co-amplification are highlighted in blue, with EGFR- and PDGFRA- focally amplified tumors denoted red and green, respectively. (**b**) mRNA expression levels of EGFR and PDGFRA in the same cases demonstrates that receptor co-expression at the mRNA level is common in the absence of coamplification. (**c**) FACS measured total EGFR (y-axis) and PDGFRA (x-axis) protein expression of 11 primary GBM tumor sphere lines with indicated genotypes; at least 5 cases (ii, iii, v, vi, xi) show distinct cell subpopulations defined by RTK levels (xii) EGFR and PDGFRA negative control, Ba/F3 cells. Blue – Unstained cells, Red contours – EGFR-FITC or EGFR-AF647 and PDGFRA-PE double-labeled cells.

Analysis of TCGA glioblastoma data demonstrates that at least 4% (16/434) of tumors harbor explicit co-amplification of EGFR and PDGFRA (Fig. 1a), however a much larger fraction of tumors show co-expression of the corresponding mRNAs at comparable levels (Fig. 1b). Moreover, in an independent MSKCC GBM sample cohort, 37% of samples (n = 225) with high level immunopositivity for phospho-EGFR also showed strong PDGFRA protein expression (Supplementary Table S1 and Supplementary Fig. 1a). We examined the cell-to-cell distribution of EGFR and PDGFRA protein expression in glioblastoma derived lines of different genotypes by dual FACS. Only one cell line was documented to harbor EGFR/PDGFRA co-amplification (TS753^9^). All lines were assessed by array-CGH and, with the exception of TS753, showed no evidence of dual amplification of EGFR and PDGFRA loci (complete genotype information of GTS-lines used in this study is provided in Supplementary Table S2). In contrast to commercially available GBM lines, which exhibit uniform EGFR and PDGFRA expression profiles (Supplementary Fig. S1b) lines derived from surgically resected primary GBM presented with a variety of receptor expression distributions as shown in Fig. 1c. Naturally occurring and distinct subpopulations defined by protein expression levels of both RTKs were observed in lines TS600 (Fig. 1c-ii), TS543 (Fig. 1c-iii), TS12017 (Fig. 1c-v), TS603 (Fig. 1c-vi) and TS13127 (Fig. 1c-xi). We next investigated whether novel signaling mechanics emerge in the context of variation in EGFR and PDGFRA co-expression.

### Functional transactivation of PDGFRA by EGF-ligand and heterodimerization of EGFR and PDGFRA occurs in glioblastoma lines of diverse genotypes

**Figure 2:**
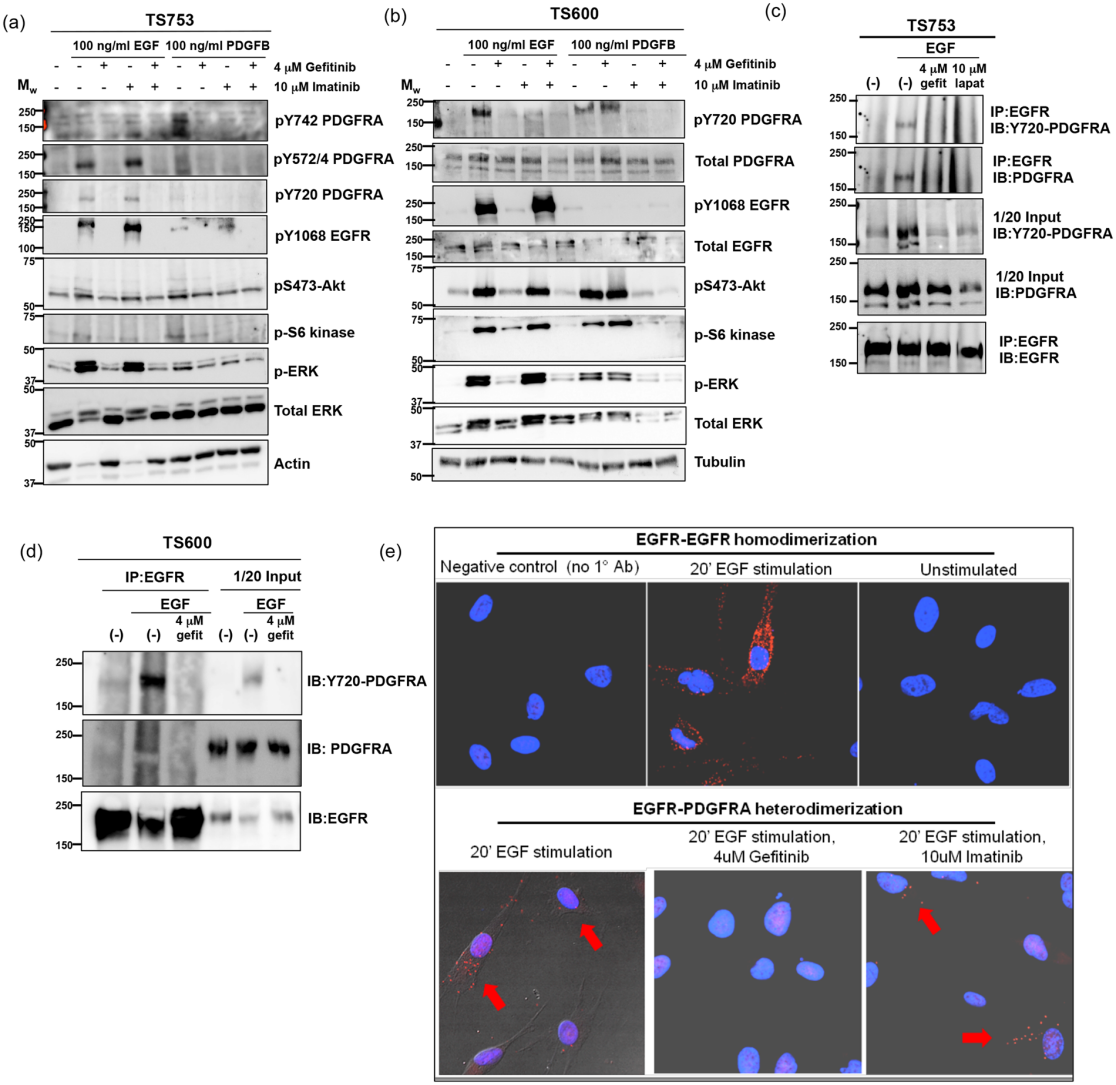
EGF ligand stimulates EGFR-PDGFRA functional transactivation and heterodimerization in glioblastoma lines of varied genotype. Tumorsphere lines of indicated genotypes were serum-starved overnight followed by next-day treatment for 4 hours with the specified RTK-inhibitors. Cells were then ligand stimulated (as indicated) for 20 minutes. Whole cell lysate were collected and 30 micrograms of protein was run on SDS-PAGE and analyzed by western blot with the indicated antibodies. EGF stimulates PDGFRA phosphorylation, which is reversed by gefitinib and, to a lesser extent, PDGFB elicits detectable phosphorylation of EGFR in all lines: (**a**) TS753 – EGFR and PDGFRA focal co-amplification and (**b**) TS600 – EGFR focal amplification and Chromosome 7 gain (also refer to Supplementary Fig. S2). (**c**,**d**) GTS-lines were serum starved overnight, treated for 4 hours with the indicated inhibitors and then stimulated for 20 minutes with 100 ng/ml EGF. Cells were subsequently lysed and 2 mg of whole cell lysate were immunoprecipitated with total-EGFR antibody overnight. Beads were washed with lysis buffer, heat-denatured, run on SDS-PAGE and probed with activated and/or total PDGFRA antibodies in tumorsphere lines (**c**) TS753, and (**d**) TS600. EGF induced co-immunoprecipitation of EGFR with activated PDGFRA (pY720) in TS753, TS600. Coimmunoprecipitation between EGFR and PDGFRA was reversed by gefitinib and, for TS753, lapatinib as well. Reverse IP with PDGFRA antibody was less efficient but also demonstrated EGFR co-IP reversed by gefitinib (Supplementary Fig. 3a). Coimmunoprecipitation between EGFR and activated PDGFRA (pY720) was also observed in EGFR-vIII positive TS12017 (Supplementary Fig. 3b). (**e**) *In situ* Proximity Ligation Assay using proximity probes against EGFR and PDGFRA was performed in co-amplified tumor sphere line TS753. Eight-chamber slides seeded TS753 cells were serum starved overnight followed by 4-hour treatment with the indicated inhibitors. Cells were stimulated with EGF for 20-minutes. Cells were counter stained with DAPI (blue) to visualize the nucleus and red-dots show fluorophore expression due to proximity of oligo-tagged EGFR and PDGFRA antibodies associated with EGFR and PDGFRA interaction. Treatment of cells with gefitinib resulted in loss of the EGFR/PDGFRA interaction and thus a loss of *in situ* PLA signals (red dots), but treatment with imatinib did not.

We previously reported the unexpected de-phosphorylation of PDGFRA at the Y720 site by EGFR inhibitors gefitinib or lapatinib in an EGFR/PDGFRA co-amplified and co-expressing line TS753^9^. Here, we observed that EGF-ligand stimulated PDGFRA at Y720 in GTS-lines of different genotypes and this was reversed by EGFR inhibitors gefitinib, lapatinib and cetuximab (Fig. 2a,b and Supplementary Fig. S2).

EGFR has been reported to heterodimerize with c-Met^18^ and PDGFRB^19^. Additionally PDGFRA has been shown to heterodimerize with FGFR1^20^. To investigate whether direct EGFR-PDGFRA receptor interaction may occur in glioblastoma cells we used co-immunopreciptation with/without EGF-stimulation to measure whether ligand-induced heterodimerization occurred in the co-amplified tumor sphere line TS753 (Fig. 2c), EGFR-amplified line TS600 (Fig. 2d and Supplementary Fig. 3a) and EGFRvIII-expressing line TS12017 (Supplementary Fig. S3b). Phosphorylated PDGFRA was pulled down with EGFR only in the condition of EGF stimulation in all GTS-lines tested, and this interaction was inhibited by EGFR inhibitor gefitinib (Fig. 2c,d). Consistent with this data, receptor interaction was also observed using proximity ligation assay^21^ with/without EGF-stimulation (Fig. 2e). We found that EGFR-PDGFRA heterodimerization (Fig. 2e, lower-left panel) occurred to a lesser extent compared to EGFR homodimerization (Fig. 2e, upper-center panel) as indicated by block arrows. Receptor heterodimerization was inhibited by gefitinib (Fig. 2e, lower-center panel) but not imatinib (Fig. 2e, lower-right panel).

### Baseline heterogeneous RTK-expression levels effect cell-to-cell variability of downstream signaling responses to targeted RTK-inhibitors

**Figure 3:**
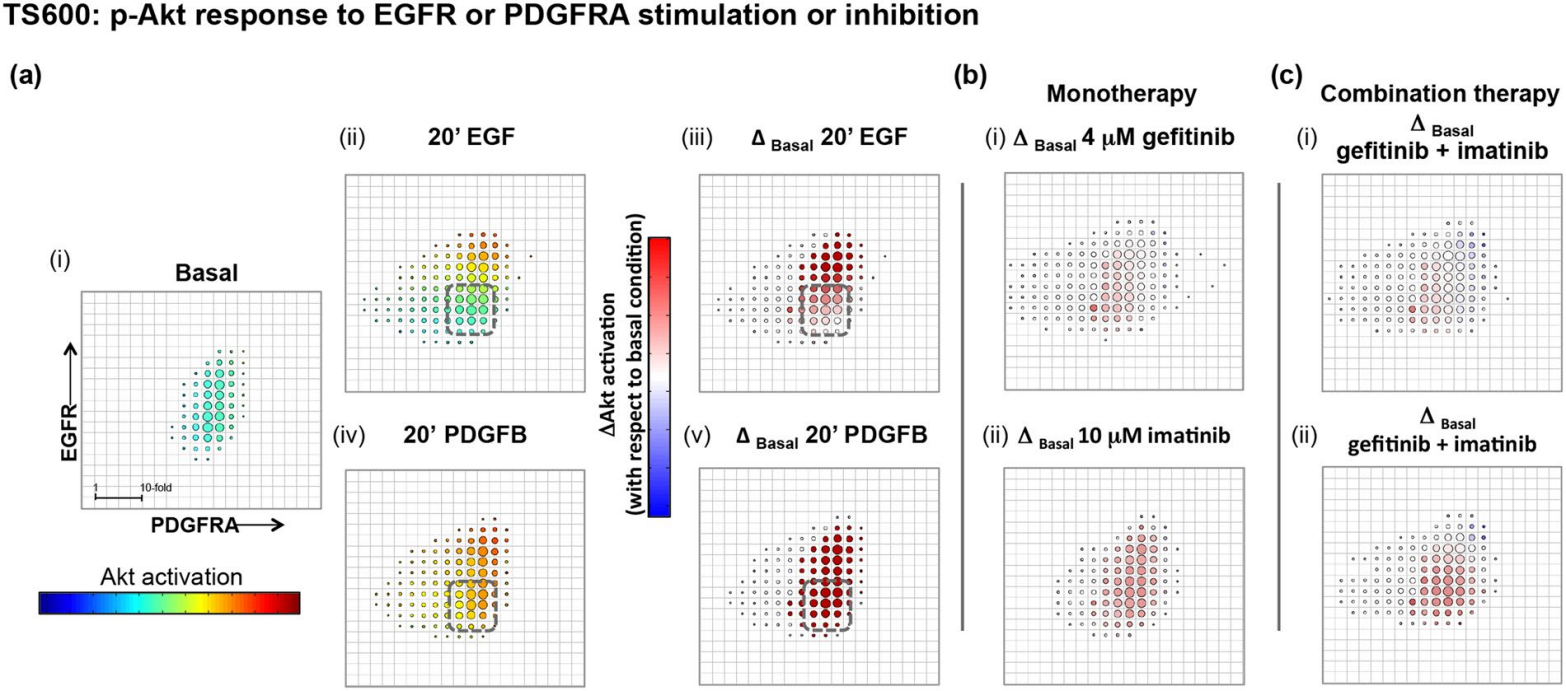
Balance of RTK expression determines tumorsphere p-Akt response to ligand and pathway inhibitors. Multichannel p-FACS experiments in TS600 were analyzed to generate heatmaps of p-Akt activation as a function of EGFR and PDGFRA expression. Mean normalized p-Akt levels for cells in each bin are represented by a rainbow color map, and changes in mean from basal levels are represented by blue/white/red color map with a scale of +/− 10-fold change (scale bar shown in “Basal” condition), where red and blue cell bins in the presence of RTK inhibitor indicates more resistant and sensitive cell subpopulations, respectively. Circle size corresponds to number of cells per bin. (**a**) Ligand effect on p-Akt. (i, ii iii) EGF stimulates p-Akt along a gradient of EGFR expression and appears to have little effect on cells harboring low-level receptor expression (dashed box). EGFR and p-Akt levels were positively correlated (ii, iii: correlation coefficient = 0.72) (i, iv, v) PDGFB stimulates p-Akt in all cell bins. The subset of cells with low EGFR expression was responsive to PDGFB ligand at the concentrations tested. (**b**) Mono- and (**c**) combination RTK inhibitor treatment effect on p-Akt: Treatment with either (i) 4 μM gefitinib or (ii) 10 μM imatinib or (c) dual inhibition in the presence of ligand reduced p-Akt levels to basal levels or below in cells with higher RTK expression but failed to inhibit low-expressing subpopulations.

We next investigated downstream signaling effects of variable EGFR and PDGFRA expression using multi-channel FACS to measure p-Akt or p-ERK signaling responses to ligand and/or targeted inhibitors as a function of RTK expression cell-to-cell. We grouped cells into bins defined by EGFR and PDGFRA expression and calculated median p-Akt or p-ERK signal for each bin. To determine the effect of ligand or targeted therapy on p-Akt relative to basal i.e. the net p-Akt signal, we calculated the change in median p-Akt signal between basal and treated conditions  per bin (this “delta analysis” was also performed for p-ERK, see Materials and Methods). Relative fluorescence intensities (RFIs) per EGFR-, PDGFRA- and p-Akt or p-ERK antibody-conjugated fluorophores were normalized for each experimental condition and corrected for cell size by removing covariance with forward- and side-scatter channels. The effect of this normalization accounts for the difference in the distribution for TS600 shown in Fig. 1c.ii, where there are two subpopulations defined by EGFR expression, and in Figs 3a.i and 4a.i where the subpopulations appear less distinct.

**Figure 4:**
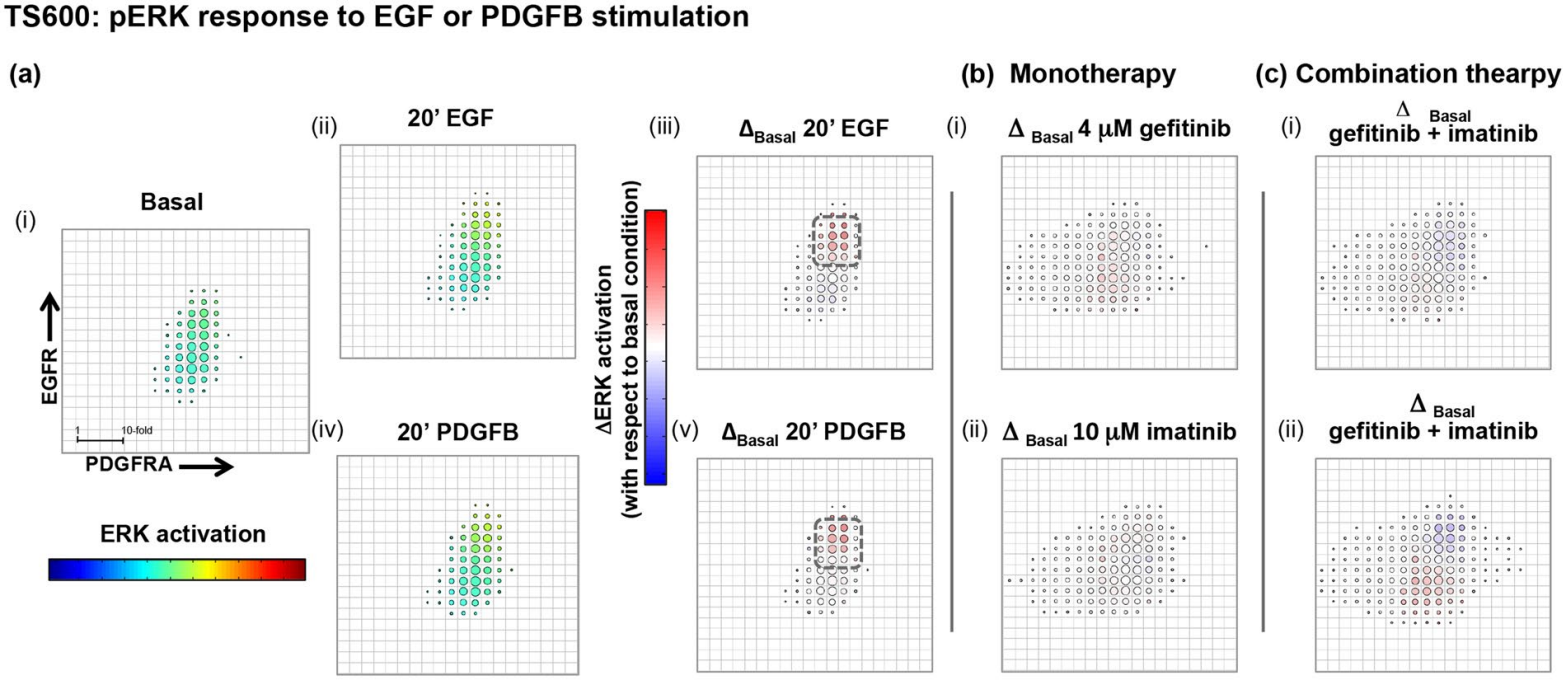
Balance of RTK expression determines tumorsphere p-ERK response to ligand and pathway inhibitors. Phospho-ERK activation was analyzed as a function of EGFR and PDGFRA expression and plotted as in Fig. 3. (**a**) Ligand effect on p-ERK. (i – v) EGF and PDGFB stimulate p-ERK only in cells harboring high levels of both RTKs (cell-bins in dashed squares) and activation patterns are similar for both ligands. (**b**) Mono- and (**c**) combination RTK inhibitor treatment effect on p-ERK: Treatment of TS600 with either (i) gefitinib or (ii) imatinib or (**c**) dual inhibition in the presence of ligand demonstrates a similar pattern of mixed response among cell populations defined by RTK expression, with greater inhibition seen in populations with higher EGFR and PDGFRA co-expression.

Positive correlations between EGFR and p-Akt levels were observed in EGF-stimulated p-Akt response patterns for both GTS-lines TS600 (Fig. 3a.ii,iii: correlation coefficient = 0.72) and TS12017 (Supplementary Fig. S4a: correlation coefficient = 0.46) indicating that cells with higher levels of EGFR also evidenced stronger p-Akt response. In contrast, PDGFB (100 ng/ml) induced Akt phosphorylation in all cells with a weak correlation with PDGFRA in TS600 (Fig. 3a.iv,v Correlation coefficient = 0.26). In TS12017, PDGFB selectively stimulated cells harboring high relative levels of PDGFRA (Supplementary Fig. S4b). For TS600, we compared the pattern of p-Akt and p-ERK response as a function of RTK level. Interestingly, stimulation of p-ERK by either EGF or PDGFB ligand only occurred in the fraction of cells with high relative levels of both RTKs (Fig. 4, dashed squares).

Treatment of GTS-lines with EGFR and PDGFRA inhibitors effectively reversed ligand-stimulated p-Akt and p-ERK levels overall, but different sensitivities were discernible when cells were grouped by RTK expression. Neither monotherapy with EGFR- or PDGFRA-targeted inhibitors (Figs 3b and 4b; Supplementary 4), nor their combination (Figs 3c and 4c) were able to inhibit p-Akt or p-ERK signal below basal levels in all cells, and in TS600 the most resistant populations were those with the lowest RTK expression levels. We also tested p-Akt response to a selective pan class I PI3K inhibitor BKM-120^22^. Contrary to the pattern seen with RTK inhibition cell fractions with relatively low RTK levels were more sensitive to the PI3K inhibitor (Supplementary Fig. S5).

### Baseline heterogeneous RTK-expression levels effect cell-to-cell variability of cell growth inhibition by targeted RTK-inhibitors

**Figure 5:**
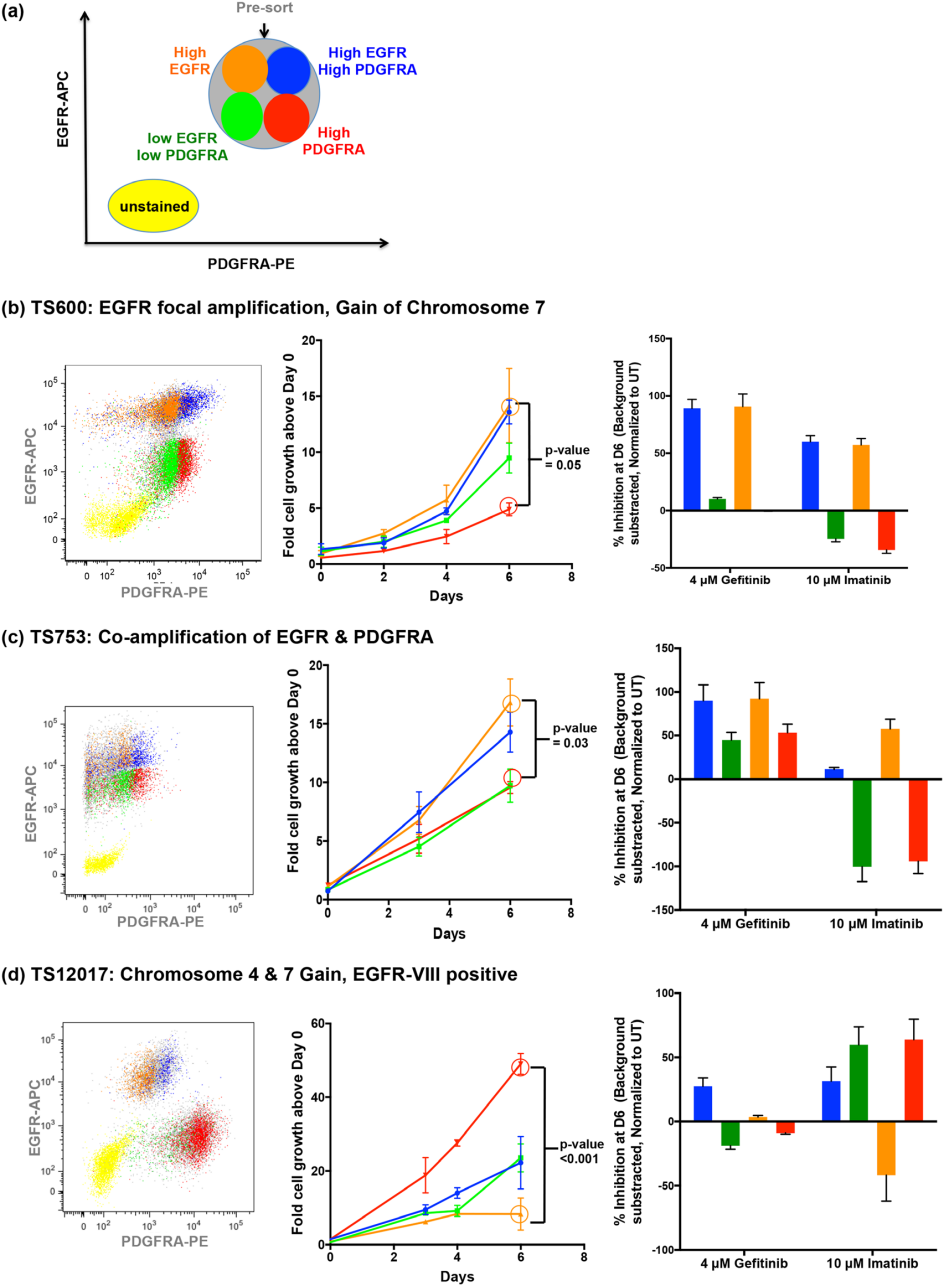
Heterogeneity of response to targeted therapies occurs as a function of the balance of RTK expression in primary GBM samples. (**a**) Patient derived glioblastoma lines were viably sorted for EGFR and PDGFRA surface immunopositivity (cells with relatively higher EGFR and PDGFRA co-expression = blue. EGFR = orange, PDGFRA = red and cells with relatively lower EGFR and PDGFRA co-expression = green). (**b**–**d**) *Left panels:* Verification of relative RTK-expression by post-FACS re-sort analysis. *Center panels:* RTK-expression defined subpopulations were individually cultured for 6 days and their comparative growth rates were measured using the resazurin fluorometric assay. *Right panels:* Each subpopulation was treated for six days with either gefitinib or imatinib and their comparative growth were measured using the resazurin fluorometric assay on Day 6. Each bar represents measurement in triplicate, background (media only) subtracted and normalization to Day 0; (**b**) TS600, (**c**) TS753 (**d**) TS12017.

We next investigated how heterogeneous EGFR and PDGFRA expression affect cell growth rates and responses to receptor-targeted inhibitors. Three GTS lines representing the most common genotypes were sorted by EGFR and PDGFRA expression and evaluated for growth inhibition in response to treatment with either gefitinib or imatinib over a period of 6 days (Fig. 5). Subpopulation RTK levels were verified by post-sort FACS (Fig. 5b–d left panels) and western blot analysis of sorted cell fractions (data not shown). In general, untreated subpopulations expressing relatively higher EGFR and/or PDGFRA protein exhibited significantly higher growth rates compared to lower-expressing fractions in all GTS-lines examined (Fig. 5b–d center panels).

Significant differences in growth inhibition were found across sorted subpopulations from all lines (Fig. 5b–d). In both TS600 (Fig. 5b) and TS753 (Fig. 5c) cells enriched for higher EGFR expression grew faster (Fig. 5b,c center panels, blue and orange growth curves compared to green and red) and correspondingly had a higher percent growth inhibition in response to gefitinib (Fig. 5b,c right panels). Interestingly in these two lines, imatinib had higher inhibitory activity in the fractions enriched for higher EGFR, but conversely increased growth in the cells enriched for high PDGFRA expression (Fig. 5b,c right panels).

In contrast, in the EGFRvIII-expressing TS12017 GTS-line, the high-PDGFRA subpopulation had a significantly faster growth rate compared to the other subpopulations (Fig. 5d center panel, red growth curve) and imatinib showed activity in all TS12017 RTK-sorted subpopulations with the notable exception of cells enriched for higher EGFR expression (Fig. 5d, right panel, orange bar). Here, gefitinib had lower overall inhibitory activity in all cell fractions and appeared to increase growth in the cells enriched for high PDGFRA expression (Fig. 5d, right panel).

## Discussion

The prevalence of dual RTK co-amplification in adult and pediatric GBM is unique among solid tumors (though the phenomenon has been reported in a subset of intimal sarcomas^23^ and more recently in esophagogastric cancers^24^). The most common co-activated and co-amplified RTKs in GBM are EGFR and PDGFRA, and our study provides the first observation of heterodimerization and evidence of functional transactivation as a possible adaptive mechanism to promote their co-expression during gliomagenesis. Evidence of EGF-stimulated heterodimerization of these two receptors suggests a mechanism for activation of signaling pathways downstream of, and specific to, each receptor in the absence of available PDGF ligand. However, it is not yet clear whether transactivation depends on direct heterodimerization or if intermediate signaling proteins are involved.

While the EGFR and PDGFR signaling pathways have largely overlapping downstream components, PDGF is a stronger activator of PI3K subunits and of other targets such as SHP2^25^. Interestingly, we found PDGFRA-Y720 to be the most consistent site of PDGFRA phosphorylation induced by EGF and abolished by EGFR inhibitors. PDGFRA Y720 phosphorylation mediates SHP2 binding and phosphorylation at Y542 and Y580^26^. SHP2 C-terminal phosphorylation is critical for PDGF-driven ERK1/2 activation, and has been shown to be a key mediator of oligodendrocyte precursor proliferation and PDGFRA-driven gliomagenesis^27, 28^. Although SHP2 is also activated by EGFR, the mechanism is indirect via recruitment of GAB1^29, 30^ and tyrosyl phosphorylation of SHP2 by EGFR has been shown to be greatly reduced compared to PDGFRA^26, 31^. Taken together, the subcellular localization and activation of SHP2, and thereby its downstream effects, is likely dependent upon the simultaneous expression, activation and mutation states of multiple RTKs including EGFR and PDGFRA^32^.

Determining the relative contribution of EGFR/PDGFRA transactivation to glioma signaling is complicated by the prevalence and heterogeneity of expression of both receptors in gliomas. Previous reports have measured EGFR expression in glioma cell lines ranging over two orders of magnitude; a variation not associated with amplification of the locus (e.g. from 0.27 × 10^4^ receptors/cell (U-251 MG) to 1.6 × 10^6^ (D-37 MG)^33^. Our FACS analysis of three established cell lines found relatively compact distributions of immunopositivity for EGFR and PDGFRA within each line, with standard deviations less than 10-fold in range. In contrast, the ranges of surface expression for tumor sphere lines typically varied more widely cell-to-cell and, in some cases, appeared to define distinct tumor cell subpopulations. Moreover, co-expression of EGFR and PDGFRA was shown to be common even in the absence of co-amplification. Given the wide diversity of dual receptor expression patterns, we anticipated that responses to ligand and/or targeted inhibitors of one or both RTKs might be a complex function. Indeed, delta analyses in different GTS-lines revealed that *in vitro* downstream signaling responses to ligand were proportional to cellular levels of the dominantly expressed RTK. Moreover, p-Akt responses to  RTK-targeted and downstream PI3K-targeted-inhibitors also showed dependence upon both EGFR and PDGFRA protein expression. Differential effects were seen between subpopulations differing by 10- to 100-fold in receptor immunopositivity. At minimum, these findings suggest a cautious interpretation of RTK pathway addiction studied in heterogeneous tumor sphere lines. We note that intratumoral heterogeneity of EGFR and PDGFRA expression is present even in the absence of RTK amplification, and that rapid selection of subpopulations defined by expression alone may be unappreciated by the experimenter.

The combination of two or more targeted inhibitors has been suggested as necessary to effectively address the lack of tumor response due to the inherently polyclonal nature of GBM^5, 8^. However, additive or synergistic toxicities of such combinations may prevent this approach^34^. Our data suggests that simply targeting multiple RTKs fails to inhibit large cell fractions with low RTK expression. We found that viable cell subpopulations harboring low RTK-expression had the lowest Akt response to ligand. Of note, while these cells exhibited slower intrinsic growth rates relative to cells with higher RTK expression levels, they were also less responsive to high concentrations of RTK-targeted agents, even in combination. Thus through the first systematic characterization of cell-to-cell protein expression of these RTKs, our study demonstrates that a single glioblastoma TS-line has a spectrum of RTK drive that may influence downstream pathway addiction. Our study also shows that wide variation in RTK expression can exist in glioma tumor sphere model systems even in the absence of amplification, and that this variation can predict the same form of differential sensitivity to targeted inhibitors seen in amplified vs. unamplified subpopulations. The methods we developed are straightforward to apply to *in vitro* assays of drug sensitivity where the expression levels of one or more signaling proteins are hypothesized to be a factor in response.

## Methods

### TCGA data analysis

Array-CGH Level 2 and Level 3 datasets and mRNA expression data were downloaded from the public TCGA portal (Agilent CGH 244 K platform, MSKCC source site) (‘Cancer Genome Atlas Research Network’ 2013). CNA log2 ratios for EGFR and PDGFRA were determined as previously published^2^.

### Human tumor collection, tumor sphere preparation and tumor sphere culture

The experimental protocols outlined in this manuscript were approved by Memorial Sloan Kettering Cancer Center’s Institutional Review Board protocol (Protocols 99–125, 06–107). All experiments and methods were carried out in accordance with the approved guidelines. All participants signed an informed consent specifically approved for this study. Fresh human GBM tissue samples were obtained from patients who consented before surgery under an Institutional Review Board-approved protocol (Protocols 99–125, 06–107). Following de-identification and serial bank number assignment obtained tumor samples were banked as frozen tissue and used to generate tumor sphere cultures. Washed tumor specimen samples were processed as previously described^9^.

### Protein extraction, inhibitor treatment, immunoprecipitation and immunoblotting

Inhibitor treatment, whole cell lysis and protein extraction, immunoprecipitation and immunoblotting were performed as described previously^7, 9^. The molecular weight marker in the western blots shown was Precision Plus Protein Kaleidoscope (#161-0375, Biorad). Detailed methodology may be found in the Supplementary Methods online.

### DuoLink

We followed the Manufacturer’s instructions as described in the DuoLink *in Situ* Red Starter Kit (92101) by Olink Bioscience, Uppsala, Sweden using the following primary antibodies for EGFR and PDGFRA respectively: EGFR ab, EGF Receptor (EGFR1) Mouse mAb (IP Specific) #2256 and PDGFRA ab Cell Signaling, PDGF Receptor α (D1E1E) XP® Rabbit mAb #3174. Detailed specifications of our methodology may also be found in the Supplementary Methods section.

### Multichannel p-FACS

Inhibitor treated glioma cells were fixed with 1.6% paraformaldehyde in PBS, permeabilized in 150 μl of ice-cold 90% methanol in water and incubated for 20 min on ice (or stored at −20 °C until the assay was performed). Washed cells were stained with fluorophore conjugated primary antibodies in 1%BSA in PBS for 40 min. Sorting was performed on a FACSAria flow cytometer (BD Biosciences).

### Live sorting and cell viability assessment of tumorsphere lines

Inhibitor treated and Fc-receptor blocked glioma cells were washed with 1%BSA/PBS and stained with EGFR-Alexa647 and PDGFRA-PE. Cells/fraction were lysed for immunoblotting analysis or plated at 2000 cells per well in a 96-well plate for resazurin (#BUF012A, AbD Serotec as part of Biorad, Hercules CA) assessment of cell viability as per manufacturer’s instructions.

### Delta analysis

We exported and normalized FACS-signal (measured in relative fluorescence units, RFIs) per fluorophore per experimental condition by removing covariance with forward- or side-scatter channels as surrogates measures of cell size. Cells were grouped into bins defined by a range of EGFR and PDGFRA RFIs. Median p-Akt (or p-ERK) RFI of cells in each bin was calculated by a custom analytic package developed in R as follows:

$$\frac{{\rm{Net}}\,{\rm{experimental}}\,{\rm{effect}}\,{\rm{on}}\,{\rm{p}} \mbox{-} \mathrm{Akt}/{\rm{bin}}}{({\rm{relative}}\,{\rm{to}}\,{\rm{basal}})}={\rm{mean}}\,\mathrm{treatment} \mbox{-} \mathrm{derived}\,{\rm{p}} \mbox{-} {\rm{Akt}}/{\rm{bin}} \mbox{-} {\rm{mean}}\,{\rm{basal}}\,{\rm{p}} \mbox{-} \mathrm{Akt}\,{\rm{signal}}/{\rm{bin}}$$

### Population sensitivity fitting and ScatterSlice

ScatterSlice analysis to generate EC50 and –Vmax data were performed as described previously^35^. The fitting algorithm uses the Hessian matrix to estimate errors on the fitted parameters. Detailed methodology of population sensitivity fitting and ScatterSlice analysis may be found online in the Supplementary Materials.

### Growth assays measuring drug sensitivity of sorted populations

After being sorted cells were plated at a concentration of 2000 cells/well in a 96-well plate in growth medium described in section “Tumor Sphere Preparation”. To determine cell viability we used alamarBlue cell viability reagent (resazurin) (DAL1025, Invitrogen, Carlsbad, CA). Specifically for our assays, we measured absorbance after 4 h of cell incubation with resazurin following alamarBlue protocol for several days during the course of a week. In the case where cells were treated with inhibitors cells were plated at same concentration in growth medium containing the inhibitors that were going to be assayed. No additional medium was added during the course of any of the assays.

## Electronic supplementary material

Supplementary Information
