# Supplementary material for: EGFR and PDGFRA co-expression and heterodimerization in glioblastoma tumor sphere lines
Source: Sci Rep. 2017 Aug 22;7:9043. doi: 10.1038/s41598-017-08940-9 (PMC5567352; doi:10.1038/s41598-017-08940-9)
Supplement: Supplementary file 1 — Supplementary Information [file 41598_2017_8940_MOESM1_ESM.doc]

**EGFR and PDGFRA co-expression and heterodimerization in glioblastoma tumor sphere lines.**

**Debyani Chakravarty^1^, Alicia M Pedraza^2,3^, Jesse Cotari^4, 5, 6^, Angela H Liu^7^, Diana V Punko^8^, Aushim Kokroo^9^, Jason T Huse^2,3,10^, Gregoire Altan-Bonnet^4,5^ and Cameron W Brennan^2,3,11^**

^1^Kravis Center for Molecular Oncology, Memorial Sloan Kettering Cancer Center, New York, NY 10065, USA

^2^Human Oncology and Pathogenesis Program, Memorial Sloan Kettering Cancer Center, New York, NY 10065, USA

^3^Brain Tumor Center, Memorial Sloan Kettering Cancer Center, New York, NY 10065, USA

^4^ImmunoDynamics Group, Programs in Computational Biology and Immunology, Memorial Sloan Kettering Cancer Center, New York, NY 10065, USA

^5^Center for Cancer Systems Biology, Memorial Sloan Kettering Cancer Center, New York, NY 10065, USA

^6^Department of Immunology, Weill Cornell Graduate School of Medical Sciences, New York, NY 10065, USA

^7^School of Medicine, University of California San Diego, 9500 Gilman Drive, MC 0602, La Jolla, CA 92093, USA

^8^New York Medical College, School of Medicine, 40 Sunshine Cottage Rd, Valhalla, NY 10595, USA

^9^NYU School of Medicine, 550 1st Avenue, New York, NY 10016, USA

^10^Department of Pathology, Memorial Sloan Kettering Cancer Center, New York, NY 10065, USA

^11^Department of Neurosurgery, Memorial Sloan Kettering Cancer Center, New York, NY 10065, USA

RUNNING TITLE: RTK co-expression and heterodimerization in GBM tumor sphere lines.

KEYWORDS: Glioblastoma, EGFR, PDGFRA, Gefitinib, Imatinib

**Corresponding author:**

Cameron W Brennan, MD

Memorial Sloan Kettering Cancer Center

1275 York Avenue

New York, NY 10065

United States

Tel: +1 212 639 8268

Email: [brennac2@mskcc.org](mailto:brennac2@mskcc.org)

**SUPPLEMENTARY INFORMATION**

**Figure S1. EGFR and PDGFRA protein co-expression in glioblastoma. (a)** **Representative immunohistochemistry analysis of a patient tumor sample (M753) from the MSKCC cohort**. Sample shown scored 1+ for both p-EGFR and Total PDGFRA (refer to Table I). **(b) Commercially available GBM lines exhibit uniform distributions of EGFR and PDGFRA expressions.** Dual-labeled FACS measures total EGFR (y-axis) and total PDGFRA (x-axis) in 3 different commercially available GBM lines. All three lines display uniform distributions of EGFR and PDGFRA expression. Blue-Unstained cells, Red- EGFR-AF647 and PDGFRA-PE double labeled cells.

(a)

**
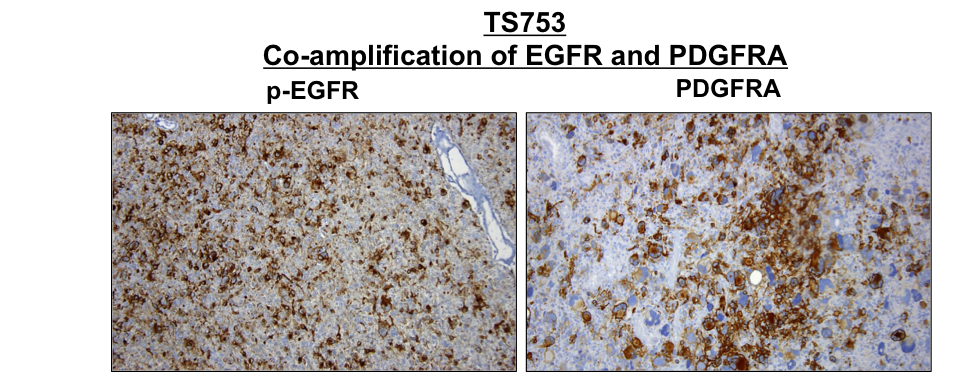
**

(b)

**
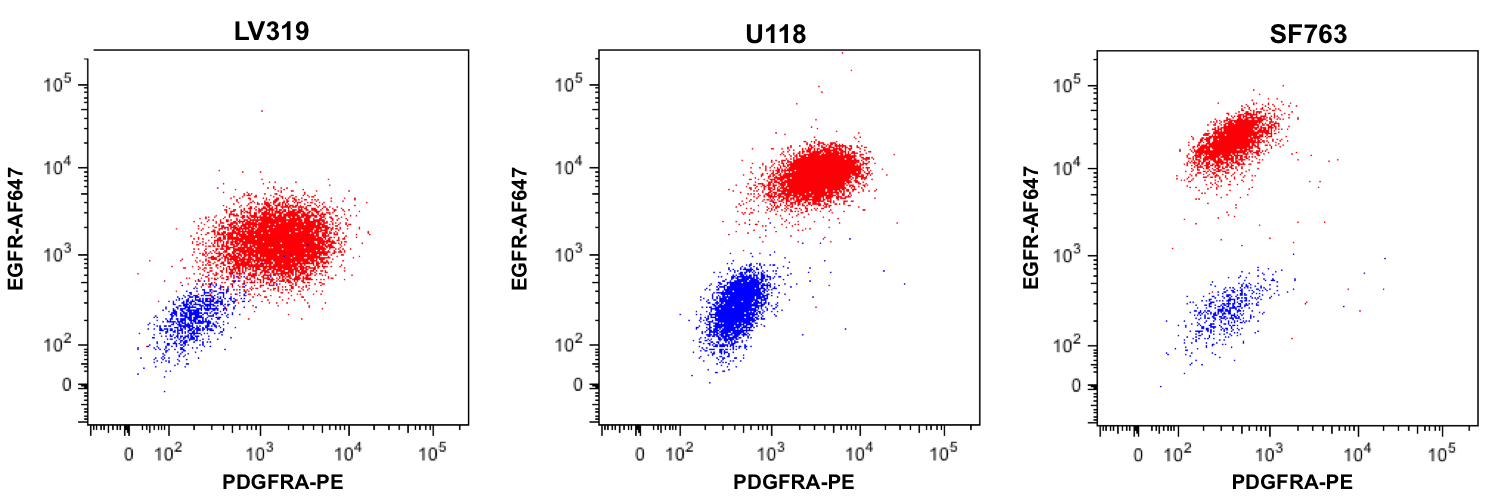
**

**Figure S2. EGF ligand stimulates EGFR-PDGFRA functional transactivation in glioblastoma lines of varied genotype which may be inhibited by different classes of EGFR inhibitors.** Tumorsphere lines of indicated genotypes were serum-starved overnight followed by next-day treatment for 4 hours with the specified RTK-inhibitors. Cells were then ligand stimulated (as indicated) for 20 minutes. Whole cell lysate were collected and 30 μg protein was run on SDS-PAGE and analyzed by western blot with the indicated antibodies. EGF stimulates PDGFRA phosphorylation, which is reversed by gefitinib and, to a lesser extent, PDGFB elicits detectable phosphorylation of EGFR in all lines: **(a) TS12017 – Chromosomes 4 &7 gain, and EGFR-VIII positive** and **(b) TS12046 – Chromosome 7 gain and chromosome 10 loss.** **(c) Western blot of whole-cell lysates from serum starved co-amplified TS753 treated with either lapatinib (10 μM) and/or PTK787 (10 μM)** **4h before activation by specified ligands.** 30 μg of lysates were run on SDS-PAGE and analyzed with Western blot using the specified antibodies. EGF stimulates Y720-PDGFRA activation and lapatinib reverses this effect. **(d) Western blot analysis of whole cell lysates from serum starved TS600 or TS12017 treated with 100 nM cetuximab, 4 μM gefitinib or 10 μM Imatinib before activation with 100 ng/ml EGF.**  30 μg of lysates were run on SDS-PAGE and analyzed with Western blot using the indicated antibodies. Similar to 4 μM gefitinib, 100 nM cetuximab inhibits EGF stimulated Y720-PDGFRA phosphorylation in both TS600 and TS12017.


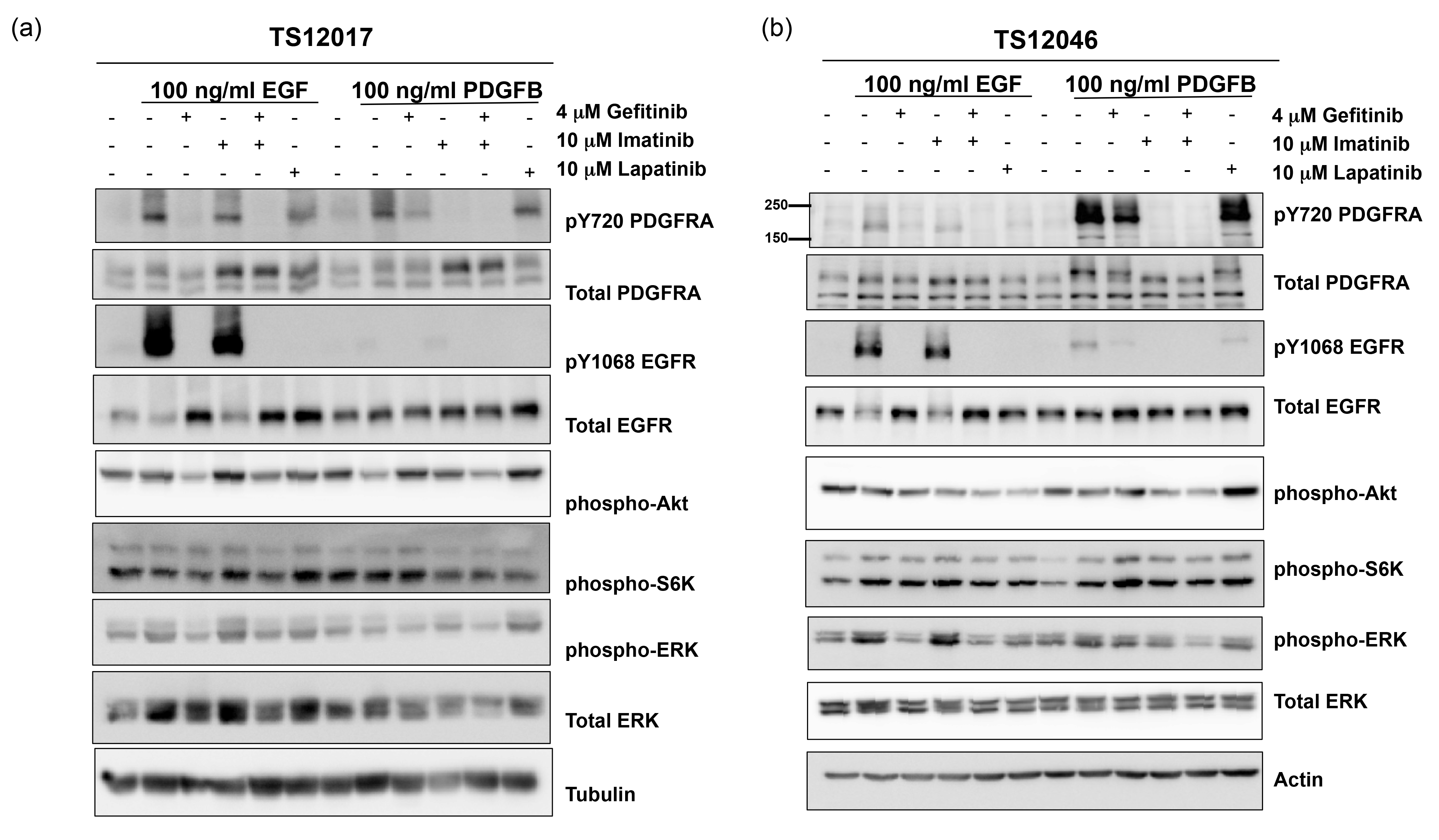
 **
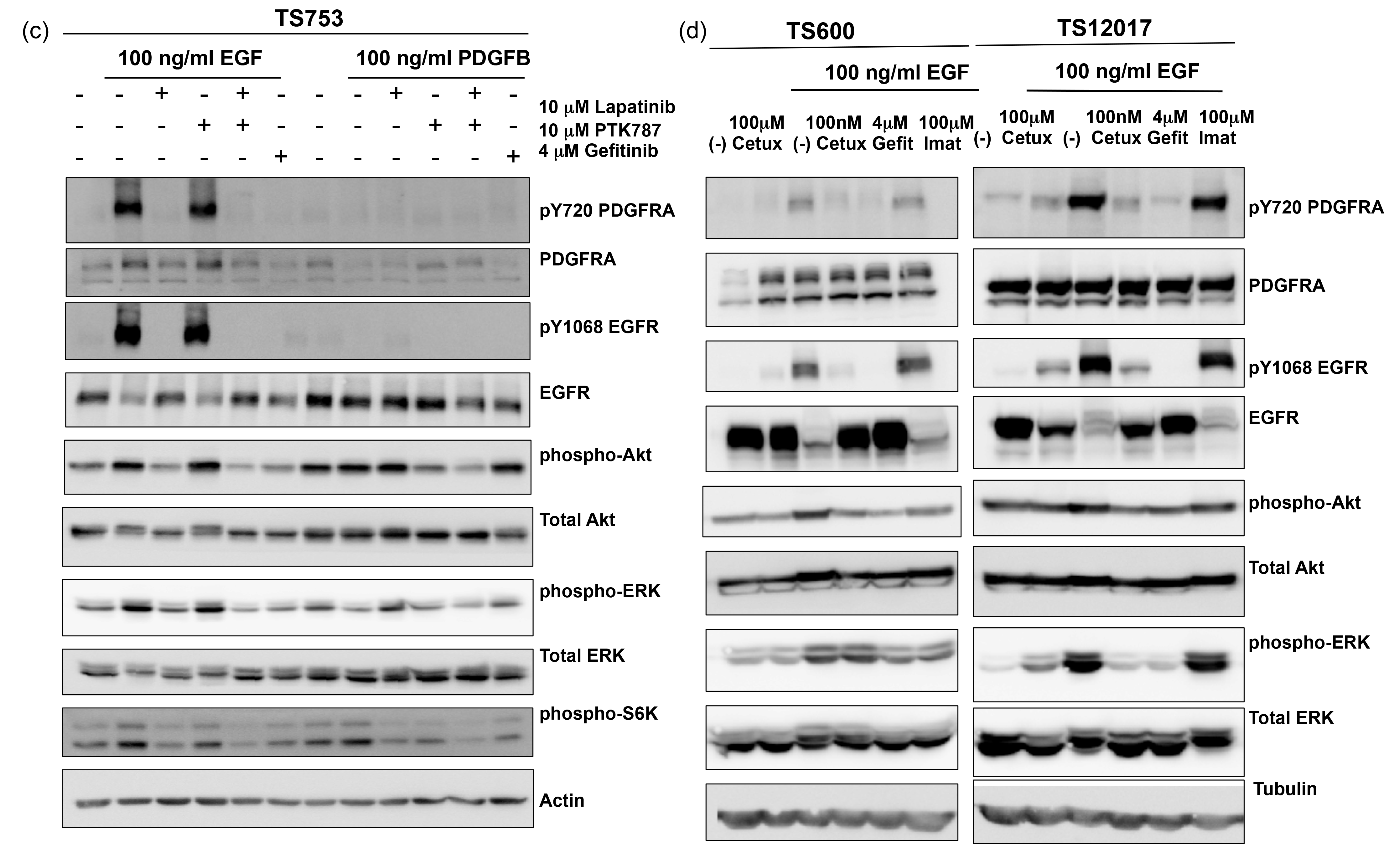
**

**Figure S3. Preliminary data suggests EGF ligand stimulates reciprocal EGFR-PDGFRA heterodimerization** (a) Reverse IP with PDGFRA antibody was less efficient but demonstrated that PDGFRA was able to reciprocally pull down EGFR and this was also reversed by gefitinib in TS600 and (b) Glioblastoma tumorsphere TS12017 was serum starved overnight, treated for 4 hours with the indicated inhibitors and then stimulated for 20 minutes with 100ng/ml EGF. Cells were subsequently lysed and 2 mg of whole cell lysate were immunoprecipitated with total-EGFR antibody overnight. Beads were washed with lysis buffer, heat-denatured, run on SDS-PAGE and probed with activated and/or total PDGFRA antibodies in tumorsphere lines. (c) In situ Proximity Ligation Assay using proximity probes against EGFR and PDGFRA was performed in co-amplified tumor sphere line TS753. Eight-chamber slides seeded TS753 cells were serum starved overnight followed by 4-hour treatment with the indicated inhibitors. Cells were stimulated with EGF for 20-minutes. Cells were counter stained with DAPI (blue) to visualize the nucleus and red-dots show fluorophore expression due to proximity of oligo-tagged EGFR and PDGFRA antibodies associated with EGFR and PDGFRA interaction. Shown here are high-resolution color separated micrographs of two separate fields in the EGF-stimulated, EGFR-PDGFRA heterodimerization condition of the proximity ligation assay.


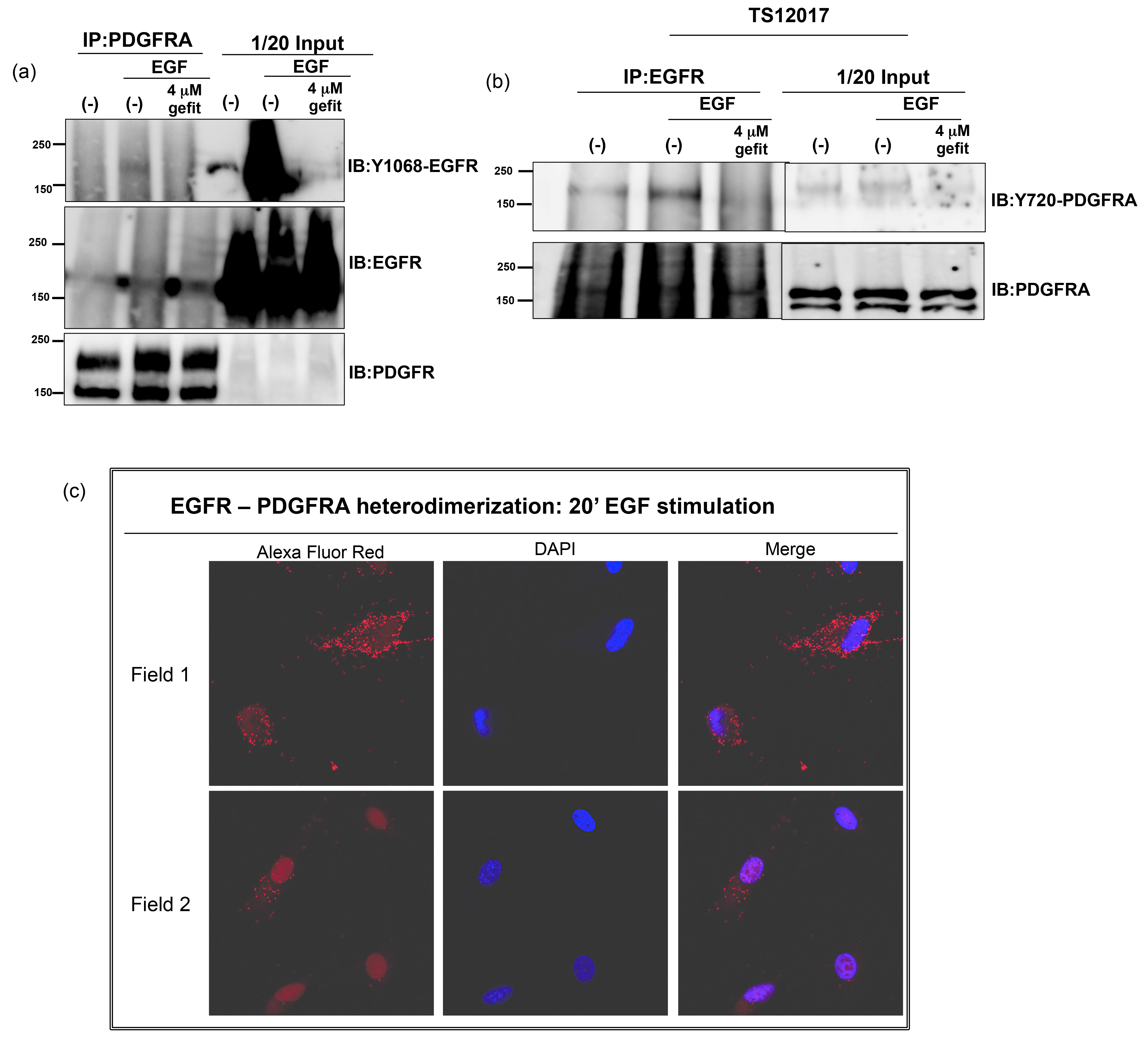


**Figure S4. Balance of RTK expression determines tumorsphere response to ligand and/or pathway inhibitors.** Multichannel p-FACS experiments in TS12017 were analyzed to generate heatmaps of p-Akt activation as a function of EGFR and PDGFRA expression. Given that Akt is basally phosphorylated in all GBM lines we have thus far tested we evaluated p-Akt inhibition relative to basal as an indicator of *in vitro* drug potency. Mean normalized p-Akt levels for cells in each bin are represented by a rainbow color map, and changes in mean from basal levels are represented by blue/white/red color map with a scale of +/- 10-fold change (scale bar shown in “Basal” condition). Circle size corresponds to number of cells per bin. **(a) EGF effect on p-Akt.** EGF stimulates p-Akt in all cell bins (red circles indicate activated p-Akt above basal condition) and neither gefitinib nor lapatinib as monotherapy inhibit p-Akt below basal levels (near-complete absence of blue-signal in any cell bins). **(b) PDGFB effect on p-Akt.** PDGFB stimulates p-Akt along a gradient of PDGFRA expression (gradient of blue to red circles along PDGFRA x-axis, correlation coefficient = 0.39) and 10μM imatinib does not suppress p-Akt activation in the presence of PDGFB (near-complete absence of blue-signal in any cell bins).


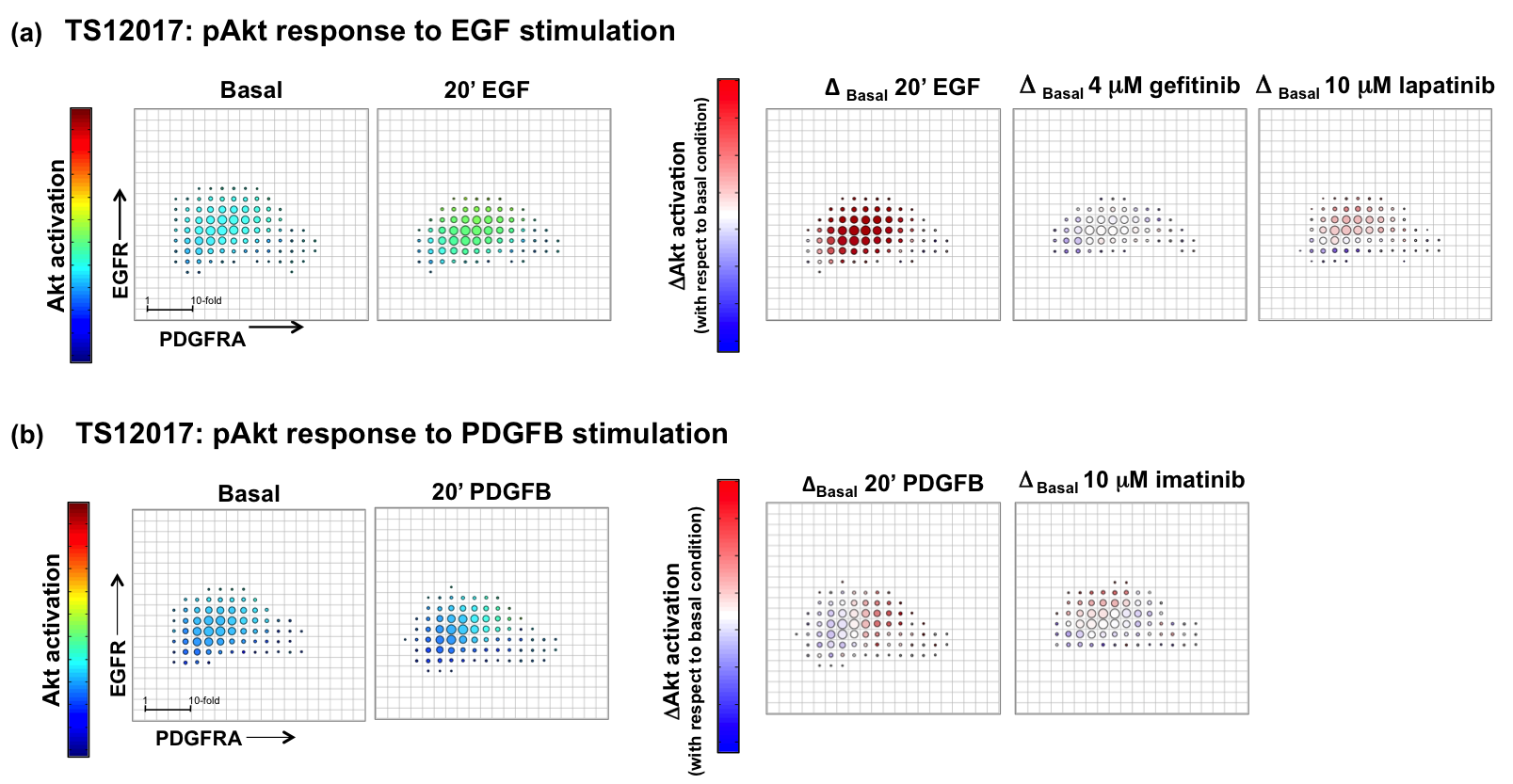


**Figure S5. Balance of RTK expression determines tumorsphere response to pan-PI3K inhibitor BKM-120.** **(a)** Delta analysis of PDGFB-stimulated TS753 **(b)** At highest concentration of BKM120 used (2 μM), only cells with low levels of both RTKs show p-Akt inhibition below basal. Cells with high levels of either EGFR or PDGFRA show persistence of p-Akt signal.

**
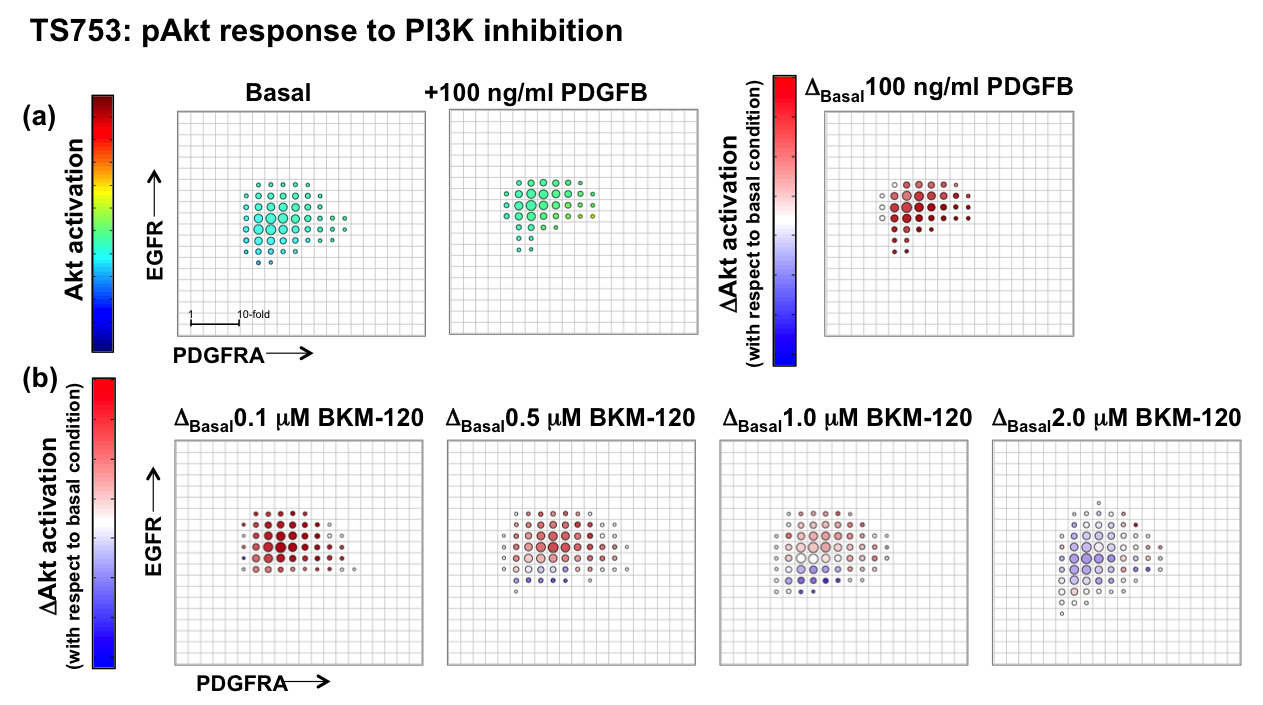
**

**SUPPLEMENTARY TABLES**

**Table SI. Immunohistochemical analysis of p-EGFR and total PDGFRA co-expression in patient glioblastoma tumors.** From a MSKCC patient-cohort, 225 formalin fixed tissue samples were stained with p-EGFR and total PDGFRA antibodies. Slides were scored by a neuropathologist as highly positive (2+), moderately positive (1+) or absent (0) for p-EGFR and/or Total PDGFRA and tabulation of total number of cases is shown. Thirty-seven percent of patient-tumors exhibit co-expression of Total PDGFRA and activated EGFR (highlighted in red).

**
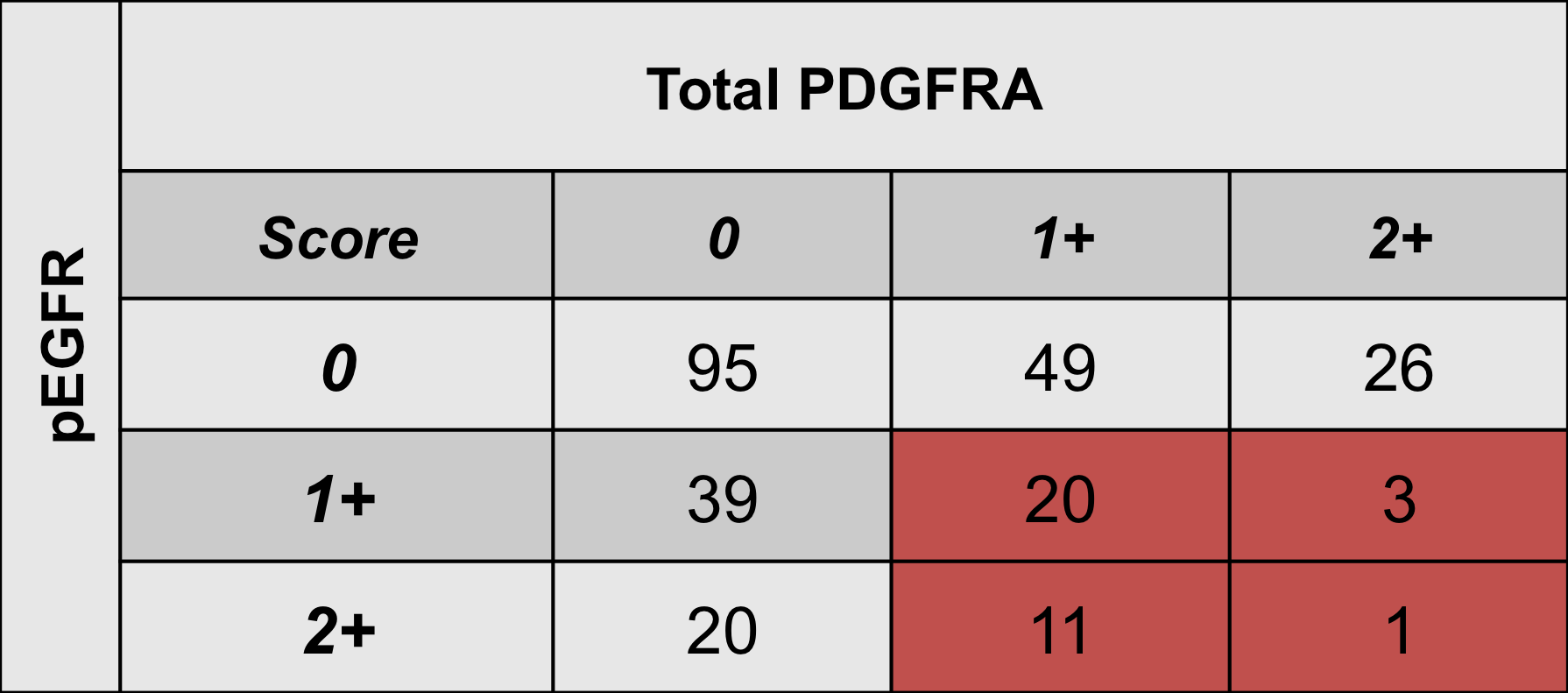
**

**Table S2. Array CGH determined genotypes of GTS lines used in this study.** GTS line DNA was isolated from all patient-tumor derived GTS lines and Level 2 data (normalized) for Agilent 244k aCGH data (MSKCC) were downloaded and parsed into to subsets of probe values. CNA focality, a measure of how many genes are included in simple and complex aberrations, was scored in each sample using a Genome Topography Scan method previously described (GTS [5, 43, 49]).


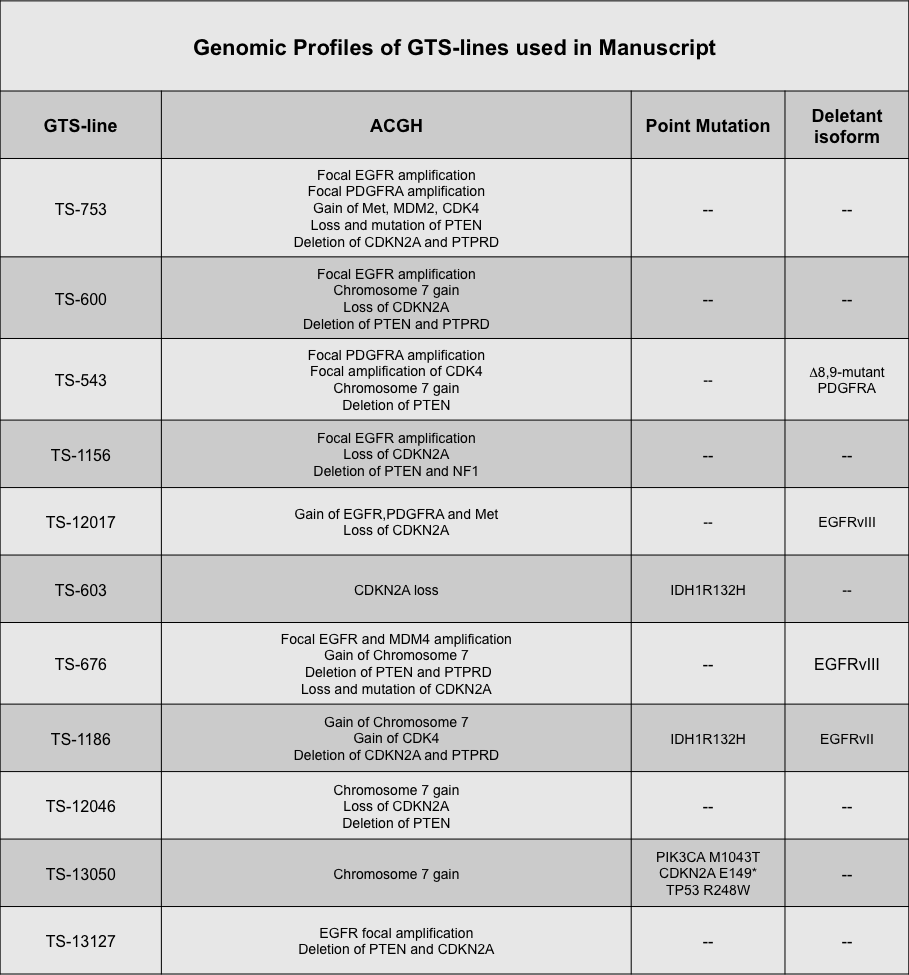


**SUPPLEMENTARY EXPERIMENTAL PROCEDURES**

***Antibodies***

All antibodies were purchased from Cell Signaling Technology, Beverly, MA unless otherwise indicated. For immunoblotting: p-Akt (#4060), Akt (#9272), p-EGFR (pY1068) (#3777), EGFR (#4267), p-PDGFRA (Y754) (#2992), p-PDGFRA (pY849)/PDGFRB (pY857) (#3170), p-p42/44 MAPK (pT202/pY204) (#9101, #4370), p44/42 MAPK (Erk1/2) (#9107), and p-p70 S6 kinase (pT389) (#9234). Where indicated the following antibodies from Sigma-Aldrich, St. Louis, MO were used for immunoblotting α-tubulin (#T9026), p-PDGFRA (pY-742) (#P8246), and β-actin (#A5441). p-PDGFRA (pY720) (sc-12910), PDGFRA (sc-338), Santa Cruz Biotechnology, Santa Cruz, CA.α-tubulin (#05-829), Millipore, Billerica, MA. For immunoprecipitation: Membranes were probed with p-EGFR (pY1068) (#3777), p-PDGFRA (pY720) (sc-12910) and total EGFR (#4267) and PDGFRA (sc-338 Santa Cruz). For Duolink: PDGFRA (#3174), and/or EGFR1 mouse mAb (IP-specific Cat#2256) and/or EGFR rabbit antibody (#4267). For simultaneous measurements of PDGFRA, EGFR and p-Akt or p-ERK, the following antibodies were used: PDGFRA-PE conjugated (sc-8533), Santa Cruz Biotechnology, Santa Cruz, CA; EGFR-Alexa488 (#5616), p-Akt-Alexa647 (#4075), and p-p42/44 MAPK-Alexa647 (T202/Y204) (#4284), Cell Signaling Technology, Beverly, MA. For live sorting: EGFR-Alexa647 (sc-120 AF647, Santa Cruz Biotechnology, Santa Cruz, CA) and PDGFRA-PE conjugated (#8533, Cell Signaling Technology, Beverly, MA)

***Inhibitor treatments and immunoblotting***

Tumor cells were grown in standard growth medium without growth factors for 18 hours, followed by 4h incubations with gefitinib, imatinib or both; a negative control was incubated with an equivalent amount of DMSO. After drug incubations, cells were activated with 100ng/ml EFG and/or 100ng/ml PDGFB as indicated. Incubations in presence of ligand were left for 20min before harvesting the cells. Cell pellets were flash frozen in liquid nitrogen. Cell pellets were lysed on ice for 20 min in CelLyticTM MT Mammalian Tissue Lysis/Extraction Reagent (Sigma-Aldrich, C3228) supplemented with protease inhibitor cocktail Complete Mini EDTA-free (Roche, 04 693 159 001) and phosphatase inhibitor cocktail PhosSTOP (Roche, 04 906 837 001). Lysates were centrifuged to 13,000rpm for 15min, and supernatant were saved for further analysis. Samples were separated by SDS-PAGE and transferred to nitrocellulose membrane for immunoblotting. Antibodies used in immunoblotting and immunopreciptiation experiments are listed in the Supplementary Materials and Methods section.

***Immunoprecipitation.*** Cells were placed in media without growth factors the day before immunoprecipitation. On the same day, 100 μl of protein A/G PLUS agarose beads (Santa Cruz Biotechnology) were blocked with 1% BSA/PBS for 2h at 4^o^C, washed 3 times with PBS and incubated overnight with either EGFR1 mouse mAb (Cell Signaling IP specific, Cat#2256), or PDGFRA (Cell Signaling Cat#3174) antibodies. The next day, antibody-conjugated beads were washed 3 times with lysis buffer, spun down at 2600 rpm. Growth factor starved cells were incubated for 4 hours with the indicated inhibitors and then stimulated with EGF for 10 minutes. Cell lysates were harvested as described above and 27 μl of antibody-conjugated bead slurry was added to 2 mg of each IP sample. Volumes of each IP reaction were brought up to a total volume 200 μl with lysis buffer and subsequently incubated overnight at 4^o^C on a rotating platform. Next day, beads were washed 3X with ice-cold PBS, spun down at 2600 rpm for 1 min each time and washed with 1ml/sample of lysis buffer. Loading buffer was added after the last wash, samples were boiled for 5 min at 95^o^C and run on a SDS-PAGE gel.

***Duolink***

Briefly, three days prior to carrying out the assay, 8x10^4^ cells were plated into each chamber of two 8-chamber slides. The day before Duolink assay execution, cells in 8-chamber slides were growth factor starved for 18-20 hours. On the day of the assay, indicated inhibitors were added to the appropriate cell-chamber for 2 hours and cells were stimulated with 100 ng/ml EGF. Ligand stimulation was arrested by aspirating the EGF-supplemented media and washing the cells twice with ice-cold PBS. Cells were fixed for 10 minutes in 300 μl BD Biosciences Cytoperm/Cytofix solution (BD Biosciences Pharmigen, Cat#51-2090KZ). Fixed cells were washed 3 times with 1X BD Perm/Wash buffer (BD Biosciences Pharmingen Cat#51-2091KZ) for 2 minutes each. Cells were blocked for 1 hour with 2% BSA/0.3% Triton X in PBS at room temperature, followed by a 1-hour incubation of cells at room temperature with primary antibodies (EGFR ab, EGF Receptor (EGFR1) Mouse mAb (IP Specific) #2256, PDGFRA ab Cell Signaling, PDGF Receptor α (D1E1E) XP® Rabbit mAb #3174) in primary antibody solution diluent (1%BSA/0.3%TritonX in PBS). Duolink protocol was then executed as per manufacturer’s instructions. Slides were dried overnight at room temperature, mounted with DAPI mounting buffer and visualized using confocal microscopy.

***Delta analysis.*** To obtain concomitant measurements of endogenous EGFR and PDGFRA abundances with downstream p-Akt/p-ERK signaling responses to ligand and/or targeted inhibitors at single-cell resolutions, we first triple-labeled GTS lines with antibodies to EGFR, PDGFRA and either p-Akt or p-ERK, each with distinct fluorophores and ran phosphoflow with fixed, permeabilized and stained cells. Flow data would be imported into software (FloJo) and median fluorescence intensities (MFIs) would be gated to exclude signal emitted from dead cells and doublets. We next normalized FACS-measured relative fluorescent intensities (RFIs) per EGFR-, PDGFRA- and p-Akt/p-ERK antibody conjugated fluorophore for each experimental condition by removing variance in these signals attributed to deviations in forward- or side-scatter channels. Then we grouped cells into bins defined by a range of EGFR and PDGFRA RFIs and calculated median p-Akt (or p-ERK as indicated in each figure) RFI of cells in each RTK-abundance defined bin. Lastly, we determined the effect of ligand or targeted therapy on p-Akt relative to basal i.e. the net p-Akt signal by subtracting mean basal p-Akt signal per bin from mean treatment-derived p-Akt per bin and we termed this data analytic process “delta analysis”. Delta analysis was performed by a custom analytic package developed in R.

***Population sensitivity fitting and ScatterSlice.*** Population sensitivity fitting and ScatterSlice analysis were as described in the methods and Supplementary Materials of . Briefly we determined the EC50 values for given cellular populations of TS543 from flow cytometry data by fitting the dose response for the geometric mean of p-Akt as a function of the inhibitor concentration with the following equation:

p-Akt([inhibitor]) = p-Akt_low_ + p-Akt_high_ – p-Akt_low_

1 + EC50_

[inhibitor]

where p-Akt_low_ and p-Akt_high_ are the baseline and the plateau of p-Akt signals. Fits were performed with Prism (GraphPad Software). The fitting algorithm uses the Hessian matrix to estimate errors on the fitted parameters. For ScatterSlice analysis, initial analysis of flow cytometry data was performed with FlowJo software (TreeStar). Data corresponding to single TS cells with 2N DNA content were identified and exported as text files for analysis by the processing R program ScatterSlice . Text files were imported into R and then divided into user-specified bins. Within each bin, a three-parameter Hill equation, which fits the base, amplitude, and EC50, was fit by minimizing the sum across all concentrations in a dose response (from *i = 1 to N_doses_*) of the squared difference between the log of each dose’s geometric mean fluorescence intensity of the response channel (p-Akt in our studies) (*z_i_*^obs^) minus the log of the three-parameter Hill equation (fitting base, amplitude, and EC_50_), normalized by multiplying by the number of cells in that dose (*N_i_*) divided by the p-response variance (*σ_i_^2^*). Errors on fitted parameters are estimated from the Hessian matrix . Tables of fitting results (values for base, amplitude, EC_50_, and their errors for different amounts of receptors) are presented as color maps or line graphs and can be further analyzed with R or exported as text files for analysis with other software.
